# DJINNI: A Novel Technology Supported Exposure Therapy Paradigm for SAD Combining Virtual Reality and Augmented Reality

**DOI:** 10.3389/fpsyt.2017.00026

**Published:** 2017-04-28

**Authors:** Maher Ben-Moussa, Marius Rubo, Coralie Debracque, Wolf-Gero Lange

**Affiliations:** ^1^Information Science Institute, Computer Science Centre, University of Geneva, Geneva, Switzerland; ^2^Experimental Clinical Psychology, Department of Psychology, University of Würzburg, Würzburg, Germany; ^3^Neuroscience of Emotion and Affective Dynamics Lab, Swiss Center for Affective Sciences, Department of Psychology, University of Geneva, Geneva, Switzerland; ^4^Department of Clinical Psychology, Behavioural Science Institute, Radboud University Nijmegen, Nijmegen, Netherlands

**Keywords:** social anxiety disorder, social phobia, VRET, exposure therapy, virtual reality therapy, augmented reality

## Abstract

The present paper explores the benefits and the capabilities of various emerging state-of-the-art interactive 3D and Internet of Things technologies and investigates how these technologies can be exploited to develop a more effective technology supported exposure therapy solution for social anxiety disorder. “DJINNI” is a conceptual design of an *in vivo* augmented reality (AR) exposure therapy mobile support system that exploits several capturing technologies and integrates the patient’s state and situation by vision-based, audio-based, and physiology-based analysis as well as by indoor/outdoor localization techniques. DJINNI also comprises an innovative virtual reality exposure therapy system that is adaptive and customizable to the demands of the *in vivo* experience and therapeutic progress. DJINNI follows a gamification approach where rewards and achievements are utilized to motivate the patient to progress in her/his treatment. The current paper reviews the state of the art of technologies needed for such a solution and recommends how these technologies could be integrated in the development of an individually tailored and yet feasible and effective AR/virtual reality-based exposure therapy. Finally, the paper outlines how DJINNI could be part of classical cognitive behavioral treatment and how to validate such a setup.

## Introduction

In the well-known movie Amélie (1), the eponymous heroine has trouble managing social situations. When a friend of hers is publicly embarrassed by his boss, being called a vegetable, she intends to help, but does not know how to react. However, her wishful thinking of having someone whispering the perfect response to her at the right time magically comes true, and she sovereignly masters the situation by replying: “You’ll never be a vegetable. Even artichokes have hearts!.” While the dream of being helped out unobtrusively when caught off-guard or feeling insecure in social situation is usually not fulfilled and may even seem naïve, the aim of this paper is to explore the technical possibilities in achieving just this. By using the metaphor of a “DJINNI,” an Arabian mythological spirit that can be summoned to help it’s “Master” and has the ability to hide itself to others, we convey our core idea behind a set of technological solutions for helping individuals fearing social situations.

The last two decades saw the emergence of virtual reality (VR) as a therapeutic tool for treatment of various mental disorders (2). Especially in the field of anxiety disorders, the immersive experience that VR offers has made it a useful tool for exposure therapy, the gold standard in the treatment of these conditions. In fact, in situations where the confrontation with the feared object or situation is expensive (e.g., fear of flying) or difficult to provide (e.g., high buildings in rural areas when treating fear of heights; fear of open spaces; audiences for fear of speaking in public, etc.), virtual reality exposure therapy (VRET) has become a tangible solution (3). Most challenging in that respect, however, is social anxiety disorder (SAD). The nature of social situations that SAD patients fear is heterogeneous. Simulating various *in vivo* situations in VR where patients can experience possible negative evaluation by others is quite difficult to achieve technologically. A lot of efforts and resources would be required to build virtual environments that would simulate different situations experienced by the patient. In fact, the heterogeneous nature of situations experienced by SAD patients is quite difficult to achieve also for traditional approaches of exposure therapy. While the presence of the therapist in traditional exposure therapy for first guidance or modeling purposes is usual in the treatment of other anxiety disorders (e.g., specific phobia or agoraphobia), it seems rather odd to accompany a patient to speeches they have to give, or to parties, or dates. As a mental disorder, SAD is desperately in need of novel technological therapeutic solutions that can overcome the current limitations. The aim of the present manuscript is to explore the potential of new emerging, alternative technologies, that have advanced a lot in the last years [e.g., augmented reality (AR) glasses and various Internet of Things (IoT) devices such as smartwatches, sporting sensors, etc.], as support tools for exposure therapy. With “DJINNI,” we propose a technological solution integrating VR, AR, and IoT technologies to deliver a complete support solution for patients suffering from SAD as well as for their therapists.

## SAD as a Mental Disorder

Social anxiety disorder (or social phobia) is a mental disorder accumulating in frequency (4, 5) with lifetime prevalence rates of 7–13% in Europe (6), and it belongs to the most prevalent mental disorders after depression and substance abuse (7, 8). It is a debilitating condition characterized by marked fear and embarrassment in social and performance situations and when under scrutiny by others (9). SAD typically has a chronic course if untreated (6), and becomes increasingly associated with comorbid mental problems such as depression, or alcohol abuse. In the Netherlands, mental health services for SAD cost 11,952€ per capita. Even the costs for sub-threshold SAD sum up to 4,687€, substantially more than for healthy individuals (10, 11). But, most importantly, it severely disrupts social and occupational functioning (6, 12) of individuals suffering from SAD.

Cognitive models of SAD (13, 14) suggest that socially anxious individuals (SAs) tend to interpret their own performance in social contexts and ambiguous social information surrounding them, in a threat-confirming way (15). In addition, they are believed to quickly attend to negative social stimuli, predominantly faces (16). Both of these cognitive biases are believed to fuel SAs’ subjective experiences of anxiety and reconfirm the fearful convictions they enter a situation with thereby maintaining the disorder (12, 13). Interestingly, measurable relationships with observable behavior (14, 17–20) or physiology (21, 22) have been indecisive. This is intriguing as cumulative evidence suggests that SAs *are* experienced as somewhat odd in interactions—although no systematic behavioral patterns have been identified (23, 24). With SAs being experts in looking for signs of devaluation, they will most likely sense negative signals validating their fear of negative evaluation (24). Consequently, along with attentional biases, and interpretational biases, measurement of psychophysiological indices and assessment as well as retraining of “true” social behavior in real social contexts should become part of therapeutic approaches in the future.

In sum, while evidence supports the notion that social anxiety is related to a negative interpretive and attentional biases, and elevated subjective anxiety levels (15), deviations in behavioral and physiological indices have been difficult to substantiate. One reason may be that fear is, according to Lang’s model of emotion (25, 26), reflected in three independent but interrelated systems: verbal report, fear related behavior, and patterns of somatic activation. He stresses that these systems are not necessarily synchronized. In fact, it is plausible to assume that, e.g., continuous reporting (on one’s thoughts, behavior, or physiology) will disrupt their natural patterns. Additionally, human (anxious) behavior is often guided by reflexive, automatic behavioral responses inaccessible to introspection (27, 28). When social anxiety is considered, self-report could be compromised by SAs’ proneness to social desirability effects (29), or again cognitive biases (15). Furthermore, SAs’ self-reported deficits in, e.g., social skills appear to be quite accurate in one context (social interaction) but not in another (public speaking) (30).

Another reason for these inconsistencies may lie in the fact that social anxiety is quite heterogeneous. Its specific form knows many facets such as fear of trembling, blushing, or sweating, and speaking, writing, eating/drinking, and urinating in public restrooms. Often, research participants are screened on general aspects of social anxiety, which they all share. However, stimuli or social scenarios might not be specific enough to tap into their particular fear.

Although effective treatment regimens exist, the very same heterogeneity is thought responsible for the smaller treatment outcomes and higher relapse rates when compared to exposure therapy in other anxiety disorders: the effect sizes of SAD treatments (1.16) lag behind those of the other anxiety disorders (>1.74) (31, 32) and of the 60% that remit due to treatment 40% relapse (33). In addition, cognitive behavior therapy (CBT), the gold standard in anxiety treatment, focuses on distorted cognitions and exposure to frequently avoided social situations, but readily ignores shortcomings in subtle interpersonal behavior.

Finally, replicable scientific investigation of observable social behavior in real life is hampered by the necessity to incorporate numbers of confederates. Their (non-)verbal reactions, even after extensive training, are neither completely controllable nor predictable. Physiological assessment with mobile hardware makes “normal” interaction awkward. In addition, a reliable behavioral observation demands at least two observers/film angles capable of registering, e.g., frequency and length of eye contact or physical distance between interlocutors, and a team of observers/evaluators blind to the conditions (of the participant). High-resolution measurement is impossible in real social environments, even under laboratory conditions.

With regard to exposure treatment, SAD is one of the few anxiety disorders where the therapist is usually absent during the *in vivo* exposure sessions. He or she can only rely on the subjective record of the patient’s description of the situation. They cannot assess the anxiety levels or behaviors “*in vivo*” to evaluate if the session was successful. In addition, gradual exposure is difficult to accomplish and the therapist cannot serve either as model or as helping hand, should an exposure task seem too difficult.

## Current State-of-the-Art Solutions

### Virtual Reality Exposure Therapy

To overcome the abovementioned difficulties in creating anxiety evoking, highly controllable, and replicable situations in real life, immersive virtual reality (IVR) technology and VRET has gained considerable attention in research and assessment of anxiety disorders over the last decennia (34). As analog to *in vivo* exposure, VRET to threat-evoking stimuli has proven to be an equally effective way for provoking (reflexive) threat responses in close-to-real situations and initiating habituation as prerequisite of treatment (35).

However, the difficulty to assess and address the complex pattern of SAD symptoms in VRET is reflected in a relative scarcity of studies in the field. Two meta-analyses by Opriş et al. (36) and a more recent one by Kampmann and colleagues (37) explored the efficacy of technology-assisted interventions for SAD since 1985. They list only a handful of high-quality studies on VRET in SAD. These studies yield generally positive results. In a study by Klinger and colleagues (38), patients participated in virtual conversations in a meeting room and at a dinner table, were scrutinized by, and needed to assert themselves against virtual agents. This treatment was found to be similarly effective as group CBT. Wallach and colleagues (39) found effects comparable to CBT in a public speaking task in a VR scenario while at the same time achieving smaller dropout rates. Similarly, Anderson and colleagues (40) also report significant improvement in a public speaking task in VR and no difference between virtual and *in vivo* exposure therapy. Moreover, they found the effect to be stable throughout a period of 1 year after treatment. Finally, Kampmann et al. (37) found VRET treatment effects by exposing patients to virtual speech situations, small-talk with strangers, job interviews, dining in a restaurant, having a blind date, or returning bought products to a shop. Most interestingly in this study, the therapist could adjust the number, gender, and gestures of avatars, the friendliness, and to a certain degree content of the semistructures dialogs depending on the patients’ needs, anxiety, and treatment progress. Yet, treatment evaluation was heavily based on subjective self-report questionnaires and improvements in speech performance as the only behavioral measure.

While the studies conducted so far document a promising future for VR applications in SAD treatment, they fall short on exploiting its full potential. Besides often being unpretentious in its audiovisual quality, they put, except of the Kampmann’s study, a strong emphasis on the fear of public speaking, largely neglecting the complex structure of other contexts in which SAD symptoms occur. More importantly, they fail to properly address the dissimilarities that exist between SAD patients with regard to attentional and behavioral indices. Therefore, it is advised to establish a VR treatment program that covers the complex pattern of SAD symptoms while allowing for patient-specific adaptations.

### The Potential of AR As a Complementary or an Alternative Tool to VR

Although numerous virtual scenarios exist for treatment purposes, they are generally not individually tailored and very few utilize behavioral measures available through VR technology such as amount of mimicry (41), interpersonal distance and movement speed (42), or gaze direction (43), nor combine them with physiological measures (44). In addition, AR devices would allow therapists to get a first-hand impression of their patient’s behavior in a social situation in order to individually tailor the VRET scenarios. AR could also incorporate physiological data from, e.g., smartwatches to analyze indices of anxiety in a situation, hint on friendly faces in a crowd *via* emotion recognition, and provide helpful sentences to the patient (invisible for others) should he/she be stuck in a conversation.

Taken together, utilizing IVR or AR means a great step forward in investigating the interplay between self-report, actual behavior, and physiology in social anxiety as well as their response to different aspects of the treatment or their predictive value for treatment outcome. While circumventing the methodological difficulties of assessment in real life, IVR allows investigating differences in behavior such as gaze direction, gaze duration, distance from, and movement speed toward audience/interlocutor, psychophysiology, and voice properties between high and low SAs, in a high ecologically valid setting. In addition, e.g., wearable AR glasses could be used to assess actual social behavior of SADs in real social contexts. But they could also provide cues for positive social interaction *via* emotion recognition and help out in conversations or provide soothing comments, should the heartbeat registered by peripheral devices indicate anxiety. In sum, such an integrative approach will yield the potentially most reliable source of assessment for all possible indices of social anxiety, will fill the gaps in our comprehension of their correlations and of SAD, and will lead to better treatment in the future.

### Application of Wearable AR Glasses in Mental Health

Similar to VR, AR has also been employed in mental disorders for the last two decades, however, in much smaller scale due to AR’s complexity and limitations (45). With the more recent emergence of modern AR wearable glasses, such as Google Glass™, and the maturation of this technology, AR started becoming a serious potential tool for treatment of various mental disorders. AR has been employed as assistive technology for social interaction by assisting users to identify/remember people and acquire more information about these people (46, 47). Swan and colleagues (48) proposed a system for brain training using wearable AR glasses. AR has also been used in treatment of small animal phobia (49, 50) with some promising results. McNaney and colleagues investigate the employment of Google Glass™ wearable glasses as an assistive everyday device for people suffering from Parkinson’s disease (51). Wearable AR glasses are recently also being utilized as tool for supporting and teaching children suffering from autism spectrum disorder in recognizing emotions and social signals in Stanford’s Autism Glass project (52, 53) and as commercial products of the start-up company Brain Power (54).

Although AR has been used in exposure therapy, to date, no serious attempts have been published to use AR in SAD treatment, though its potential use has been speculated (45). However, early attempts at simulating patients in other fields of health sciences using wearable AR glasses can be taken as a demonstration of its feasibility (55).

## Proposed Solution

### Enhancing CBT with VRET and Augmented Reality Exposure Therapy (ARET)

**Figure 1:**
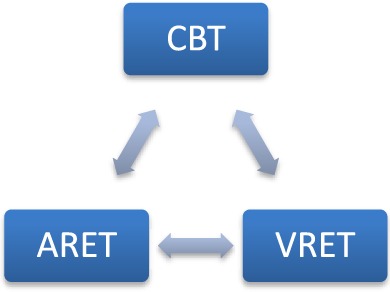
**DJINNI concept**.

To address the challenges and limitations of contemporary SAD exposure therapy approaches as described above, DJINNI is proposed as a software and hardware solution that integrates AR and VR and various sensing technologies to support SAD patient’s CBT treatment. In a nutshell, DJINNI would offer (1) an ARET that would compensate for the absence of the therapists during the *in vivo* exposure experiences and would automatically interpret various events occurring during these experiences and guide and support the patient; and (2) a VRET that would simulate exposure experiences in a safe 3D environment while incorporating the interaction and behavioral data collected from the ARET experiences. The goals and the scenarios of both ARET and VRET will be influenced by the CBT. The experiences and progress statistics collected by both ARET and VRET can influence the course of the whole SAD treatment (Figure 1).

### Augmented Reality Exposure Therapy

**Figure 2:**
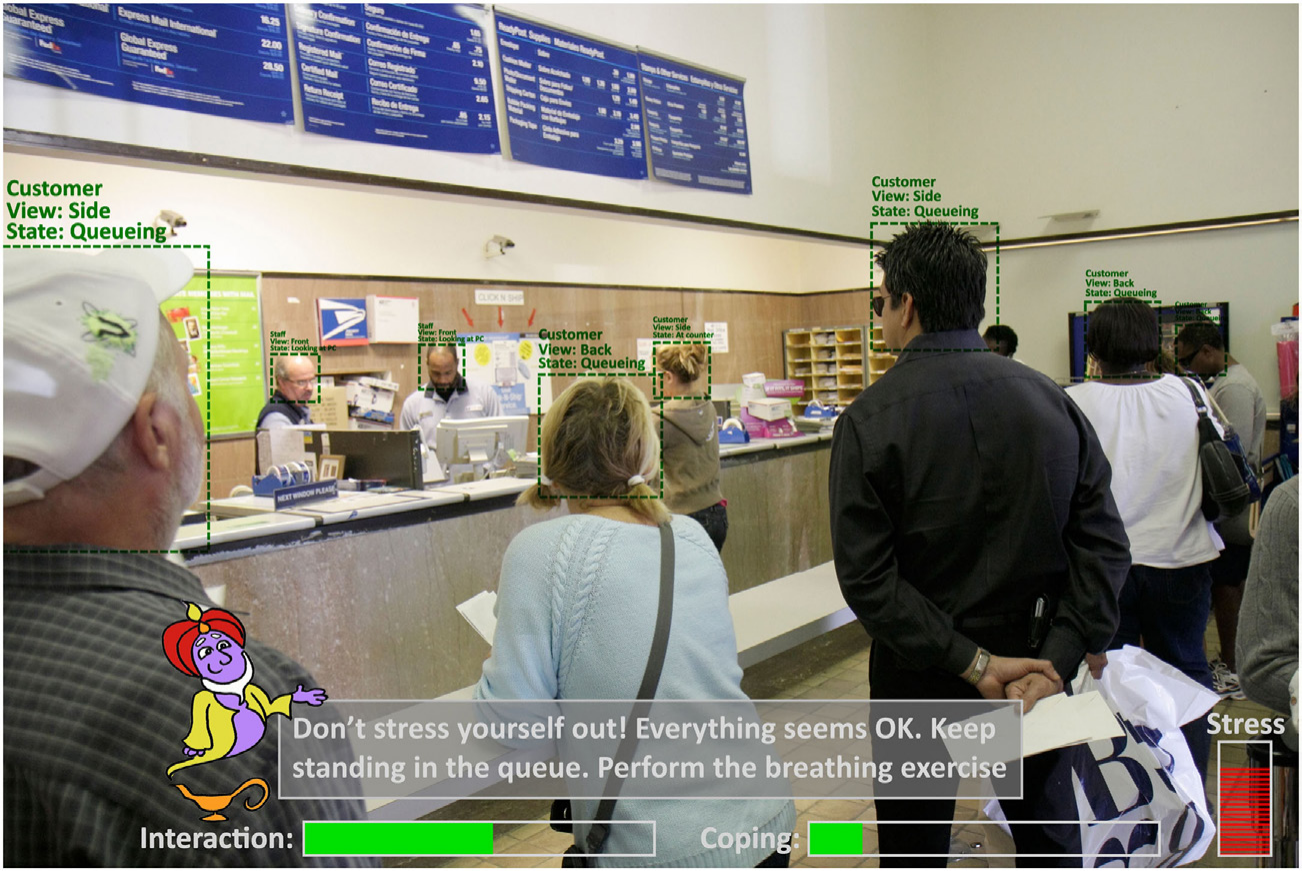
**Proposed user interface of DJINNI in *in vivo* augmented reality exposure therapy situation**. (Use of Djinni by courtesy of Gesa Kappen©; use of post-office photo by courtesy of RosaIreneBetancourt 6/Alamy Stock Photo).

In the ARET situation, DJINNI will be experienced as an intelligent assistant that guides and supports the patient during *in vivo* real-life exposure experiences. As illustrated in Figure 2, DJINNI will be experienced by the patient through her/his wearable AR glass as a system that “understands” the patient’s environment, interprets the state of the people in the environment (location, activity, conversational state, emotions, etc.), interprets the social context, assesses the state of the patient/user (location, activity, conversational state, emotions, etc.), and provides support and advices in the *in vivo* exposure experience by providing cues, advices, and soothing comments. As illustrated in Figure 2, the system’s interaction with the patient follows a gamification approach (56) where progress is measured in scores/rewards and the patient is encouraged to progress in her/his exposure treatment. The gamification approach will be partly based on previous work where techniques for visualization of rewards and progress were employed to motivate eating disorder patients (57–59).

Let us say we have a patient suffering from anxiety of being in crowded places with high probability of scrutiny by others. For the *in vivo* exposure therapy, the patient will be equipped with wearable AR glasses, a smartphone, and a wristband with physiological sensors and will be sent to, for example, a post office. For such system to function properly in an unpredictable environment, it needs to be able to reliably detect and interpret various events occurring in the environment (e.g., spatial information; people in the environment, their roles, and their current actions/behaviors; the patient, her/his actions, and her/his physiological state, etc.). With the help of the sensors, the patient is connected to and/or sensors installed in the environment, and based on the predefined information entered by the therapist about the environment, the system should be able to capture and detect various events reliably if we acknowledge some constrains and limitations as will be discussed later.

In practice, the system will understand that the persons behind the counter or person with specific shirt color and name tags are post office employees. The other persons are clients also waiting in the queue. Upon entering the post office, the system should monitor the physiological state of the patient and how she/he behaves in the environment. When hesitating, the system can display some instructional or motivational messages on the AR glasses on what to do (e.g., DJINNI could say: “*You are in the post office. Please stand in the queue and wait for your turn*”). When getting stressed the system can attempt to provide messages to calm down the patient (e.g., “*Don’t panic. Everything is ok. Please keep standing in the queue*”). When detecting the facial expressions and the gaze of others in the environment the system would be able to have a better understanding of the situation (e.g., “*The employee just greeted you and smiles at you. Try to smile back*”). The system will also be able to detect if anxiety has caused the patient toward performing inappropriate behaviors such as jumping the queue, anxiously looking people right in the eyes, or especially avoiding eye contact (e.g., “*Relax. No reason to panic. Don’t stare at her/Try to look into her eyes once a while*,” or “*Call a friend on the phone to feel better*”).

### Virtual Reality Exposure Therapy

The DJINNI VRET system will complement the ARET system by allowing the patient to re-experience similar exposure events in the safe 3D VR environment. The novelty in DJINNI’s VRET system in comparison with traditional VRET solutions lies in its incorporation of data collected by the ARET system, and, by doing so, it is possible to automatically generate and adapt VR elements to simulate the events similarly to how they occurred in the *in vivo* ARET experience. Thus, it gives the patient a platform to, first, re-experience (replay) previous experiences recorded by the ARET system. Consequently, they can learn to objectively analyze/evaluate others’ and one’s own behavior to prevent negative rumination/post-event processing. Finally, they eventually learn how to deal with these experiences in the safe environment of VR as the ARET feeds right into the VRET platform. Similar to the ARET system, the VRET system will also follow a gamification approach to motivate the patient during her/his therapy by feeding back the actual achievements but also the progress that is already accomplished.

Considering the “post-office scenario” as described in Section “Augmented Reality Exposure Therapy,” DJINNI’s VRET system would utilize the data related to the events and the people in the post office environment to automatically adapt the VRET virtual world by simulating similar density of crowd in the post office, specific events that occurred (various people starting looking at the patient, someone in the queue smiled at the patient, etc.), the timing and duration of these events, etc.

### Technical Specifications of DJINNI

**Figure 3:**
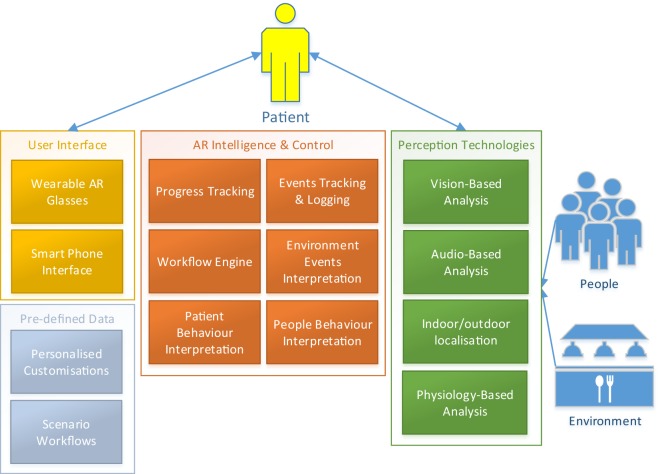
**DJINNI augmented reality exposure therapy system architecture**.

The DJINNI solution consists of two separate systems that complement each other and exchange data between each other to improve the treatment. As illustrated in Figure 3, DJINNI’s ARET system will rely on several intelligent software components that together determine the functioning and behavior of the system.

In general, DJINNI’s ARET platform consists of the following:

- *Wearable AR glasses* for displaying DJINNI’s messages to the user and capturing the events in the environment through the built-in camera.
- *Smart phone* for giving the patient a familiar and safe interface to interact with the system.
- The components *environment events tracking, patient behavior interpretation, and people behavior* analyze data acquired by the various perception components, the predefined environment information, and workflows and interpret the occurring event, the patient’s current behavior, or the behavior of other people in the environment, respectively. The perception components would detect what they perceive (e.g., woman facing patient is smiling), while the interpretation components would “understand” what it means in the current situation (e.g., the post office employee just greeted the patient with a smile).
- The *workflow engine* represents the core component of the ARET system and generates the supportive and guiding behaviors of the system by executing the predefined workflows during the exposure experience.
- *Progress tracking* component tracks the progress of the patient and communicates it with the therapist based on predefined objectives set by the therapist. This component is also responsible for the gamification-based reinforcement/motivation.
- *Event tracking and logging* logs all the events that occur during an exposure experience, which can be used to generate the VRET scenarios.
- *Vision-based analysis* represents all the components responsible for analyzing the video captured by the camera of the wearable AR glasses.
- *Audio-based analysis* represents all the components responsible for analyzing the audio and detected speech and environmental sounds.
- *Physiology-based analysis* represents the components that measure the patient’s physiological signals through wristband and other sensors.
- *Indoor and outdoor localization* represent components for determining the geographical location of the patient. Implementation of indoor localization may require equipping popular exposure therapy venues in the city (e.g., postal office, supermarket) with localization sensors that help the system in determining the exact location of the patient in an indoor environment.
- *Scenario workflows* are predefined workflows/programs defining, in a detailed way, how the system should behave during each situation in a specific exposure scenario/environment. Scenario workflows should be considered as complex but general definitions created for all the patients, while *individualized customization* are simple adaptations to these general workflows considering specific patient’s needs.

**Figure 4:**
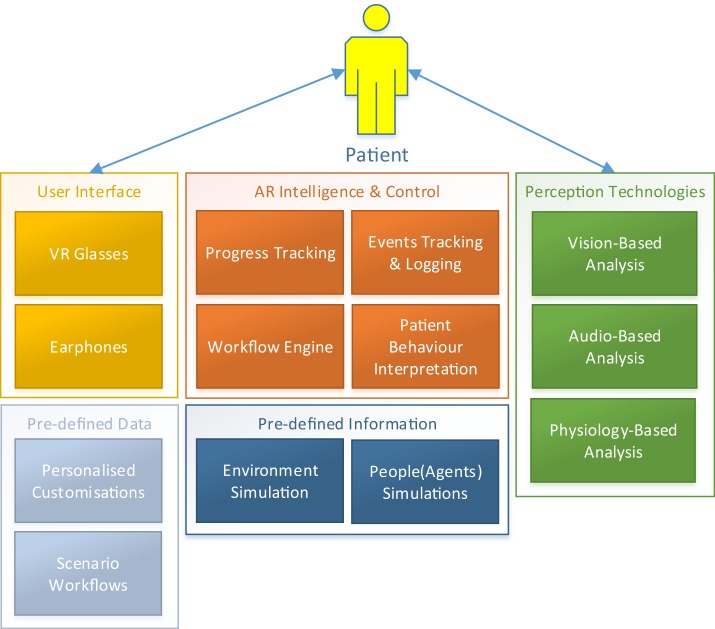
**DJINNI virtual reality exposure therapy system architecture**.

As is the case with the ARET system, the VRET system as illustrated in Figure 4 will also rely on several intelligent software components that together determine the functioning and behavior of the system. However, all the components that analyze and interpret the *in vivo* situation events are excluded as they are rather simulated instead of captured in the VR environment.

Next to the components already described above for the ARET systems, the VRET system includes the following components:

- *Environment simulation* component simulates the environment and the events in the environment based on predefined data as well as on data collected by the ARET system.
- *People/agents simulation* component simulates the behavior of the people in the scenario based on the predefined workflows as well as the by ARET captured data.

### Workflows and Interpretations

DJINNI’s scenario workflows will define step by step what actions the system should take at each possible step/situation of a specific exposure experience. Based on previous work on interactive virtual environments (60), the workflows will be implemented in artificial intelligence condition–action rules defining what actions to take given certain derived or predefined data. Table 1 illustrates the types of data that will be derived by the system or predefined by the therapist/programmer that will serve as conditions for determining the system’s actions.

**Table 1:** **What should DJINNI understand about the patient and the environment?**

	Perception technologies	Contextual information
Environment	Spatial interpretation of the environment^a^ Localization and tracking of objects in the environment^a^ Detection and interpretation of environmental sounds^a^	Predefined maps of the environment Predefined annotations of the environment
People	Detection and tracking of people^a^ Identification of people^a^ Recognition of facial expressions/emotions^a^ Recognition of gesture and posture^a^ Tracking of people’s gaze^a^ Interpretation of user’s interaction state^a^	Predefined characteristics of the roles Derived interaction history with the people Derived information about the people
Patient	Interpretation of physiological state Gesture and posture recognition Tracking of patient gaze Interpretation of patient’s interaction state	Predefined scenario workflows Predefined characteristics of the patient Derived therapeutic progress Derived history of interaction

*^a^Only for ARET. In VRET, this information is simulated*.

## Technological Feasibility

The development of the DJINNI solution would require the integration of various technologies of which some are mature and others are experimental. The following sub-sections will review these technologies and determine their current level of development and will provide recommendations on how these technologies should be integrated and utilized in the DJINNI solution.

### Wearable AR Glasses

Although, the idea of having wearable AR glasses as a communication interface for guiding and supporting the patient is innovative, it can also be seen as pitfall. The currently disconnected Google Glass™ wearable glasses (61) have been for a long time seen as the standard in wearable AR glasses. However, due to their cyborg style design, they would not be suitable for patients suffering from SAD to wear them as they would attract undesirable attention. However, the latest years also saw the development of various new wearable AR glasses that may attract less undesirable attention than Google Glass™. Currently available wearable AR glasses such as Laforge Shima™ (62) and Vuzix VidWear™ B3000 (63) will enable DJINNI to exploits the advantages of AR glasses without the negative impact risked if the technology is visible. Furthermore, the emerging waveguide optical technologies [e.g., Trulife Optics™ (64) and Dispelix™ (65)], which is also being used in Laforge and Vuzix, will enable many wearable AR glasses manufacturers to produce more fashionable and less attention-attracting glasses in the near future.

#### Consequence for DJINNI

For the first prototype of DJINNI, it is the intention to employ Laforge Shima™ or Vuzix VidWear™ B3000 AR glasses.

### Audio- and Vision-Based Perception and Interpretation Technologies

Although our perception capabilities as humans is quite advanced and we are able to easily distinguish between the different objects in our human vision and to understand the characteristics and affordances of these objects, yet, for a computer system nowadays, it is impossible to reliably detect and recognize all the objects in its view, let alone to derive their characteristics and affordances. However, for each type of object, specific algorithms have been developed and some algorithms started to reach a level of sufficient maturity (66). Using machine learning techniques, computers are enabled to emulate human cognitive abilities such as sound detection, speech recognition, image recognition, emotion recognition, and behavior analysis.

#### Speech Recognition

Although speech recognition has significant success in research using techniques such as hidden Markov models (67), practical experiments have shown that currently available speech technologies are still not reliable enough for natural human–computer interaction as most algorithms are still too sensitive to noise and accents (68). In addition, it appears that the most reliable speech recognition stems from the English language (60).

##### Consequence for DJINNI

Because of its unreliableness, speech recognition technologies will only be used in DJINNI VRET system and only if really needed. Use of speech recognition in public places will be avoided.

#### Object Recognition/Tracking

Object recognition/tracking has seen some progress in the past decades due to innovations in learning algorithms, image processing techniques, and feature extraction (69, 70). However, most object recognition and tracking algorithms only work well with objects that are near the camera view or objects that are large enough (represented by a large number of pixels in the image).

##### Consequence for DJINNI

As the camera is part of the wearable glasses, object recognition/tracking algorithms will only be able to detect objects that are in the view of the camera and close to the patient. Small objects and objects far away will not be detected by the system. Therefore, use of object recognition and tracking should be limited in the scenario workflows to large or clearly visible objects and only at certain states of the workflows (e.g., for detecting the post office counter).

#### Facial Expression Recognition

Facial expression recognition research has been ongoing for some time and has reached a convincing level of maturity. For instance, the facial action coding system, originally designed to allow human users to objectively describe facial states (71), can now be automatically processed and classified into prototypical emotional expressions using computer algorithms (72). Various research and commercial software prototypes have been designed and have reached a sufficient level of recognition.

##### Consequence for DJINNI

When speaking about emotional expressions, one has to be aware that there is no common definition of what emotions are (73) and workflows should be limited to utilizing prototypical Ekmanian emotions—the basic emotions anger, fear, sadness, disgust, surprise, and happiness, which are believed to be the most universal/stable across cultures and contexts (74). In addition, emotion detection should only occur at states of the workflows where emotions are expected to be detected and relevant. Limiting the scenario will increase the reliability. Furthermore, the system should also be able to differentiate whether someone is talking to the patient or not.

#### Sound Detection

Sound detection algorithms have reached a very convincing level of maturity in the last years. Reliable commercial and open-source products that have been released can reliably distinguish between a large variety of sounds [e.g., ROAR™ (75) and Audio Analytics™ Ltd. (76)].

##### Consequence for DJINNI

One of the reliable sound detection products can be used to detect environmental sounds that are relevant to identify certain social environments.

### VR Technologies

The last years saw a rapid emergence of high-quality and affordable commercial VR head-mounted devices [e.g., HTC Vive™ (77), Oculus Rift™ (78), Sony PlayStation VR™ (79), Samsung Gear VR™ (80), and Microsoft HoloLens™ (81)].

#### Consequence for DJINNI

All the existing products have reached sufficient quality and can be directly deployed for DJINNI VRET system.

### Physiological and Affective State Interpretation Technologies

Over the last years, various fitness wristband and chest straps have been developed and employed to track one’s fitness performance by integrating peripheral sensors for tracking of motions and heart rate [Mio LINK™ (82), Fitbit Charge HR™ (83), and Garmin Soft Strap Premium HR Monitor™ (84)]. Information derived from these affordable fitness trackers can be used to extract some information about the patient physiological state. The more professional wristband Empatica E4™ (85) has also been gaining prominence the last years in research due to its reliability and the number of sensors that it embeds. Detected sensor signals and signals changes can be directly incorporated into the workflows and can lead to system actions. Furthermore, research has also shown that heart rate variability and galvanic skin response can also be used as a good indicator of stress (86, 87). However, interpretation of psychological signals is also prune to errors due to noise and to the other causes of the same physiological changes.

#### Consequence for DJINNI

As indicated with other components, it is possible to reach a certain level of reliability if limited to certain scenario. When a change in physiological signals is preceded by events than can cause stress, there is a high chance that the patients feel stressed. By considering these indicators, DJINNI can reach a good level of reliability.

### Indoor/Outdoor Localization

Localization can refer to either indoor or outdoor localization, and it refers to locating an object or oneself in a building or outside. Various technologies for outdoor localization already exist. However, it is mainly GPS, sometimes complemented by WIFI router-based localization, that has been the most reliable until now. Indoor localization is enabled by various technologies (e.g., iBeacons, RFID, sound, infrared, ultra-wideband, etc.) (88). However, the most accurate solutions for indoor localization are the ones that combine various technologies rather than using only one (89, 90). Several mature commercial systems exist that also employ various technologies [e.g., IndoorAtlas™ (91) and AccuWare™ (92)].

#### Consequence for DJINNI

For DJINNI, it is also intended to combine various technologies for indoor localization and create a hybrid system based on previous work (93). However, indoor localization is not the most crucial component of the DJINNI solution. Although the example of the post office may require some indoor localization, many other exposure scenarios do not (e.g., public speaking anxiety, dating anxiety, etc.). For several scenarios where indoor localization is needed, local health institutes can install localization devices in some public buildings if these buildings can be used for exposure therapy (e.g., iBeacons).

## Validation Study

As shown above, VR and AR are certainly the keys to improve actual exposure therapies. To validate DJINNI and its ARET and VRET components, the following validation study needs to be conducted. The study aims to evaluate the impact that new technology has on therapeutic progress in a population with, e.g., SAD. The proposed design consists of three consecutive steps.

### Diagnostic Phase and Anxiety Evaluation

Naturally, the first step consists of thorough diagnostics of patients, by trained psychologist/therapists, by means of (structured) clinical interviews [e.g., Mini-International Neuropsychiatric Interview (94) or Structured Clinical Interview for DSM-5 (95)] and trait questionnaires [e.g., Social Phobia Scale (96, 97) or Fear of Negative Evaluation Scale (98)]. After the diagnostics, the therapist should repeatedly assess subjective reports of state anxiety in anxiety evoking situations by traditional scales [e.g., State Anxiety Inventory (99), Subjective Units of Discomfort Scale (100), or visual analog scale (101)] and/or its physiological equivalents (e.g., heart rate, skin conductance). These parameters at baseline should be assessed again in the same (formerly) anxiety evoking situations at the end of the treatment (after 12 weeks) and at follow-up (3 months after treatment) to eventually analyze treatment effects.

### ARET/VRET/Traditional Exposure Therapies

Assuming that traditional CBT as well as VRET are effective treatments of SAD (37, 102), the experimental design aims to evaluate the *added* value of adaptive VRET and ARET. Accordingly, six groups of patients, with a principle diagnosis of SAD would be necessary to combine all possible exposure treatments (see Table 2) with ARET.

**Table 2:** **Experimental conditions of the validation study**.

	No AR exposure therapy	AR exposure therapy
Traditional exposure therapy	Group 1	Group 2
Traditional VR exposure therapy	Group 3	Group 4
Adaptive VR exposure therapy	Group 5	Group 6

Based on this semi-experimental design and short-term and long-term changes in subjective experienced anxiety, behavioral and physiological indices can be used to evaluate the added value of (adaptive) VR and AR in exposure therapy. Furthermore, it will also help to assess the importance of technology-based individual tailoring/customization of treatment and evaluate the role of positive or negative feedback on the patient’s behavior.

## Conclusion and Discussion

The present article presented a conceptual design of DJINNI, a system that combines AR and VR to provide more effective exposure therapy solutions for patients suffering from SAD. Due to the meager effect sizes of traditional exposure therapies, heterogeneity of the patients’ difficulties in social interaction and the difficulty of personalization in current state-of-the-art VRET, an approach that exploits the benefits of wearable AR glasses is desperately needed.

Yet, the use of AR technology as well as various experimental technologies for environment analysis should not lead to the development of an unreliable system that will not be beneficial or even suitable for use by SAD patients in the end. However, by taking the limitations of various technologies carefully into consideration and incorporating various contextual information regarding the place, situation, and phases of the interaction when developing the solution, it would be possible to achieve a reliable levels of accuracy with the proposed technologies. A thorough evaluation of various technologies is needed before integration into analytical and therapeutic workflows. After doing so, the paper has proposed technologies that have reached a sufficient level of reliability to be considered mature enough for building an effective ARET system.

Another innovation of DJINNI is the adaptive nature of its VRET system. By incorporating data collected from *in vivo* exposure experience or even everyday social encounters, it is possible to improve and personalize VRET scenarios. Data collected during anxiety evoking events, as well as the behavior of the people in the patients’ environment, can be used to inform the behavior of the virtual environment and the people in it. It is expected that this solution can be more effective than traditional VRET solutions as it is very personalized: It simulates events that have been recently experienced by the patient in *in vivo* situations, and, yet, can take place in a “safe” therapy setting.

The final deployment of DJINNI would require the development of detailed workflows that define how the system should behave in ARET and VRET situations. This may require considerable work as each possible situation that may occur and possible actions to take by the system need to be defined in the workflows. However, by distinguishing between the general workflows and the individualized customizations, personalizing a system for each patient should not be too time consuming.

Ideally, by providing advanced, high-end technical support solutions, DJINNI could substantially improve the efficacy of CBT (for SAD) and thereby ameliorate the individuals’ suffering and societal burden considerably.

## Author Contributions

All authors listed have made substantial, direct, and intellectual contribution to the work and approved it for publication.

## Conflict of Interest Statement

The authors declare that the research was conducted in the absence of any commercial or financial relationships that could be construed as a potential conflict of interest.
